# Amoxicillin Retention/Release in Agricultural Soils Amended with Different Bio-Adsorbent Materials

**DOI:** 10.3390/ma15093200

**Published:** 2022-04-28

**Authors:** Raquel Cela-Dablanca, Ana Barreiro, Lucia Rodríguez-López, Vanesa Santás-Miguel, Manuel Arias-Estévez, María J. Fernández-Sanjurjo, Esperanza Álvarez-Rodríguez, Avelino Núñez-Delgado

**Affiliations:** 1 Department Soil Science and Agricultural Chemistry, Engineering Polytechnic School, University Santiago de Compostela, 27002 Lugo, Spain; ana.barreiro.bujan@usc.es (A.B.); mf.sanjurjo@usc.es (M.J.F.-S.); esperanza.alvarez@usc.es (E.Á.-R.); avelino.nunez@usc.es (A.N.-D.); 2 Soil Science and Agricultural Chemistry, Faculty Sciences, University Vigo, 32004 Ourense, Spain; lucia.rodriguez.lopez@uvigo.es (L.R.-L.); vsantas@uvigo.es (V.S.-M.); mastevez@uvigo.es (M.A.-E.)

**Keywords:** antibiotics, bio-adsorbents, emerging pollutants, soil pollution

## Abstract

The antibiotic amoxicillin (AMX) may reach soils and other environmental compartments as a pollutant, with potential to affect human and environmental health. To solve/minimize these hazards, it would be clearly interesting to develop effective and low-cost methods allowing the retention/removal of this compound. With these aspects in mind, this work focuses on studying the adsorption/desorption of AMX in different agricultural soils, with and without the amendment of three bio-adsorbents, specifically, pine bark, wood ash and mussel shell. For performing the research, batch-type experiments were carried out, adding increasing concentrations of the antibiotic to soil samples with and without the amendment of these three bio-adsorbents. The results showed that the amendments increased AMX adsorption, with pine bark being the most effective. Among the adsorption models that were tested, the Freundlich equation was the one showing the best fit to the empirical adsorption results. Regarding the desorption values, there was a decrease affecting the soils to which the bio-adsorbents were added, with overall desorption not exceeding 6% in any case. In general, the results indicate that the bio-adsorbents under study contributed to retaining AMX in the soils in which they were applied, and therefore reduced the risk of contamination by this antibiotic, which can be considered useful and relevant to protect environmental quality and public health.

## 1. Introduction

Emerging pollutants include a wide range of chemical compounds, such as various pharmaceutical products, and specifically antibiotics [1,2]. In 2020, in the European Union (EU), the average total consumption of anti-bacteria compounds for systemic use (ATC Group J01) was 16.4 defined daily doses (DDD) per 1000 inhabitants [3]. These compounds are not fully absorbed in the intestine, causing them to be excreted in significant amounts (up to 90%) through urine and feces [4,5], thus passing to wastewater in the case of humans, and to manure pits or manure accumulations in the case of animal farms. These contaminants can pass into various environmental compartments and may directly cause undesirable effects in soils [6], including the promotion of antibiotic resistance [7,8,9], and/or be absorbed by plants used for human or animal consumption, entering the food chain, as has been shown for lettuce and other vegetables [7,10,11].

One of the most widely used antibiotics in both human and veterinary medicine is amoxicillin (AMX), which is frequently used as a first-choice drug for the treatment of serious infections [12]. Between 80 and 90% of this antibiotic is excreted due to its poor absorption [13], then reaching the environment, and achieving concentrations of 127.49 ng L^−1^ in wastewater [14].

Different authors point out that antibiotics are frequently detected in treated wastewater, because they come from human use, but also from other sources such as agriculture and livestock production [15,16,17,18]. In this regard, one of the current strategies to alleviate water scarcity is the reuse of previously treated wastewater, which could result in public health issues due to the presence of different chemical pollutants and microbes [19]. Wastewater treatment tries to decrease nutrient loads [20] and pathogens [21], among other contaminants, but many current treatments are not sufficiently effective in retaining and inactivating pharmaceuticals such as antibiotics [22,23].

Current EU and United States (US) legislations do not include concentration limits for antibiotics in treatment plant effluents [24,25], making it more probable that antibiotics reach soils through WWTP-purified water used in irrigation [26]. In addition, antibiotics may be spread on soils through WWTP sludge used as fertilizer in agricultural crops and silvo-pastoral systems [27], and can subsequently be incorporated into the food chain.

Soils have a relevant buffering capacity and filtering potential due to the colloids present in the clay fraction and in organic matter, which help in preventing environmental pollution [28,29,30]. The dynamics followed by antibiotics in the soil depends on their physicochemical properties, as well as on those of the soil, and also on the time of application of the residual materials, as well as weather conditions [31,32,33]. Among the various processes that antibiotics can undergo in the soil environment, it is worth highlighting mineralization, degradation, volatilization, leaching, surface runoff, bioaccumulation, and adsorption.

The specific behavior of the antibiotic AMX in the soil is highly conditioned by the pH of the medium, which affects the ionization of the compound and the surface charge of the soil colloids [34,35]. In this regard, it is highly relevant that AMX has amphoteric properties due to three functional groups present in its structure: -NH_2_, -COOH, and -OH [36]. The dissociation constants (pk_a_) of a molecule indicate its ionization state as a function of pH [37]. In the case of AMX, pk_a1_ corresponds to carboxyl groups (-COOH), pk_a2_ is represented by amine groups (-NH_2_), while pk_a3_ corresponds to phenolic groups (where hydroxyl (-OH) is attached to a C atom integrated in an aromatic ring), so that, at different pH values, AMX may appear as a cation, anion, or zwitterion [38]. Specifically, at pH < pk_a1_ AMX will appear as a cation, at pH > pk_a3_ it will appear as an anion, while at pH values between pk_a1_ and pk_a3_ it will appear as a zwitterion [39].

The presence of antibiotics in soils and water that have received the spreading/disposal of wastewater and/or sewage sludge is a matter of concern [40], so different investigations have focused on the design of a variety of systems intended to its removal [41]. Several technologies have been proposed to achieve antibiotics removal during wastewater treatment, such as the use of ozone [42] or advanced oxidation [39], although these methods tend to generate unwanted toxic side-products. In contrast, adsorption is considered a rather simple and sustainable alternative [43]. In this way, commonly used adsorbents include mineral and biological materials, as well as activated carbon, with this last adsorbent being widely used, although it has a high cost and regeneration issues [44]. In view of this, there is increasing interest in using low-cost adsorbents, such as industrial waste or by-products, for which it is necessary to determine their pollutant adsorption capacity, and in particular their potential to retain/remove antibiotics [45,46].

Among them, certain residues/by-products from the food industry, such as mussel shell, and from the forestry industry, such as pine bark and wood ash, are abundant, easily accessible and low-cost, making it interesting to determine their capacity to adsorb contaminants such as antibiotics that reach different environmental compartments. These three bio-adsorbent materials could be added to the soil or used in modules specifically designed and installed in wastewater treatment plants, to minimize the risk of dispersion of these pollutants in the environment. Mussel shell, pine bark and wood ash have already been studied previously regarding their ability to retain heavy metals, inorganic anions and antibiotics from the group of tetracyclines and sulfonamides, obtaining very promising results [47,48,49,50,51,52,53,54,55,56]. There are also previous studies dealing with the adsorption of AMX present in wastewater by means of adsorbents such as wheat grain, almond shell ash, palm bark, bentonite or activated carbon [36]. However, there are no previous publications that have focused on evaluating the effects on AMX retention derived from amending crop soils with pine bark, wood ash and mussel shell. In fact, the efficacy of these bio-adsorbents to increase the adsorption of antibiotics in soils has been previously proven for sulfonamides in the case of pine bark [57], while wood ash and mussel shell showed worse results. In addition, mussel shell has been widely studied as a bio-adsorbent in soils contaminated with heavy metals [58], and wood ash has also been investigated for this purpose (for example [59]), but studies on the application of these materials in the retention of pharmaceutical products are very scarce.

In view of the above background, the present study was performed to investigate for the first time AMX adsorption and desorption on/from different agricultural soils with and without the presence of the bio-adsorbents pine bark, wood ash and mussel shell, assessing their potential to decrease the dispersion of this antibiotic, which can be considered of relevance with regard to environmental preservation and public health protection.

## 2. Materials and Methods

### 2.1. Soils and Bio-Adsorbents

Four agricultural soils, devoted to maize and vineyard cultivation, located in different areas of Galicia (NW Spain), were selected in function of their pH values and organic matter (OM) contents. All four were characterized as detailed in the Supplementary Materials. Table S1 (Supplementary Materials) shows values corresponding to soil properties., Within them, soil pH_water_ was between 5.01 and 6.04, while organic matter (OM) content was in the range 3.06–4.59%. The texture of two of the soils (soils M1 and M2) was clay loam, while it was sandy clay loam for the other two soils (soils M3 and VO).

In addition, the following materials were used as bio-adsorbents/amendments: (a) two forest by-products: pine bark (commercially distributed by Geolia, Madrid, Spain), and wood ash (from a local boiler at Lugo, Spain); (b) mussel shell (crushed at <1 mm), from Abonomar (Pontevedra, Spain). These bio-adsorbents were characterized as indicated in the Supplementary Materials, with results shown in Table S2. Some additional data regarding characteristics of these materials have been included in previous publications [55,56].

Different soil + amendment mixtures were elaborated adding the bio-adsorbents to soil samples at doses of 48 t ha^−1^, followed by 72 h of stirring at 50 rpm using a rotatory shaker, and further homogenization by means of a Retsch splitter (Haan, Germany), all this carried out at stable temperature of 25 ± 2 °C. The pH of the different soil + bio-adsorbent mixtures was analysed, with results shown in Table 1.

### 2.2. Chemical Reagents

The antibiotic AMX used (with purity ≥95%) was from Sigma-Aldrich (Barcelona, Spain), while acetonitrile (purity ≥ 99.9%) and phosphoric acid (85% extra pure) were from Fisher Scientific (Madrid, Spain), and CaCl_2_ (95% purity) was from Panreac (Barcelona, Spain). All the solutions needed for HPLC analyses were prepared using milliQ water (from Millipore, Madrid, Spain).

### 2.3. Adsorption and Desorption Experiments

AMX adsorption and desorption were studied by means of batch experiments, performed on the different soils amended with the bio-adsorbent materials, which were added to the soils in doses of 48 t ha^−1^. For this, 2 g of the soil + bio-adsorbent mixtures was weighed, then adding a volume of 5 mL of a solution with different concentrations of the antibiotic (2.5, 5, 10, 20, 30, 40, 50 µmol L^−1^), which also contained 0.005 M CaCl_2_ as background electrolyte. The resulting suspensions were shaken for 48 h in the dark, using a rotary shaker. Previous kinetic tests indicated that the 48 h period is enough to reach equilibrium (data not shown). This step was followed by centrifuging the suspensions (15 min at 4000 rpm), and by subsequent filtration of the supernatants through 0.45 µm nylon syringe filters. Finally, AMX concentration was quantified using specific HPLC-UV equipment (an LPG 3400 SD device, by Thermo-Fisher Scientific, Madrid, Spain). Details on AMX HPLC determinations are provided in Supplementary Materials. Additionally, example chromatograms are shown in Figure S1 (Supplementary Materials).

Regarding desorption, it was studied adding 5 mL of 0.005 M CaCl_2_ to the material derived from the adsorption experiments, then repeating the procedure performed for the previous adsorption phase. In all cases, triplicate determinations were carried out.

### 2.4. Data Treatment

The experimental adsorption data were fitted to the Freundlich (Equation (1)), Langmuir (Equation (2)) and Linear (Equation (3)) models [60].

(1) $$q_{e}\;{=K}_{F}C_{eq}^{n}{}_{}$$

(2) $$q_{e}=\frac{q_{m}K_{L}C_{eq}}{{1+K}_{L}C_{eq}}$$

(3) $$K_{d}\;{=q}_{e}{/C}_{eq}$$

with *q_e_* being the amount of AMX retained, which was calculated as the difference between the concentration added and that remaining in the equilibrium; *C_eq_* is the AMX concentration in the equilibrium solution; *K_F_* is the Freundlich parameter related to the adsorption capacity; *n* is a Freundlich parameter related to the degree of heterogeneity in adsorption; *K_L_* is the Langmuir adsorption constant; *q_m_* is the maximum adsorption capacity in the Langmuir model; and *K_d_* is the partition coefficient in the Linear model.

The fitting of the experimental data to the Langmuir, Freundlich and Linear models was studied by means of the SPSS Statistics 21 software (IBM, Armonk, NY, USA).

## 3. Results

### 3.1. Adsorption

**Figure 1:**
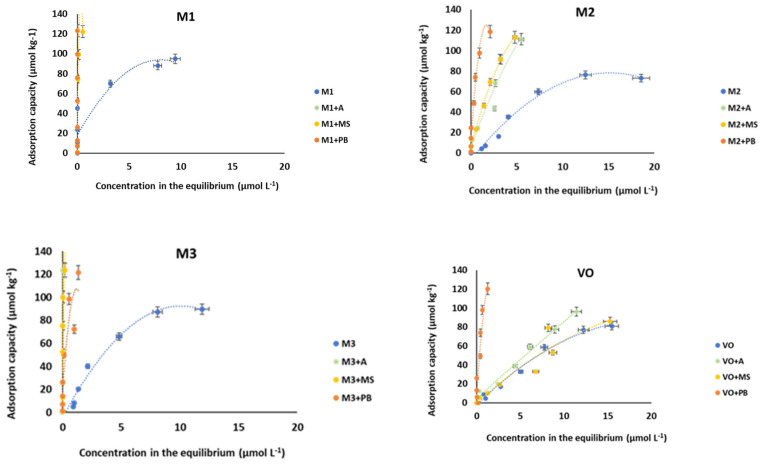
Adsorption curves for AMX in unamended and bio-adsorbent-amended soils. Average values (*n* = 3), with coefficients of variation always <5%.

As shown in Figure 1, as well as in data included in Table S3 (Supplementary Materials), pine bark performed as a very effective material for increasing AMX adsorption in the soils amended with this bio-adsorbent. Table 1 shows that the pH of each soil changes when the different bio-adsorbents are added. Specifically, pine bark (which has pH = 3.99) generally causes an acidification of the amended soil. 

Table 2 presents the values of the parameters corresponding to AMX adsorption as per the Freundlich, Langmuir and Linear models.

Considering R^2^ values, all the non-amended soils (except M1) presented an overall good fit to all three models, given that R^2^ > 0.90 for VO and M3, and R^2^ > 0.80 for M2. Focusing on both the non-amended soils and those amended with bio-adsorbents, the errors in some parameters of the Linear model, and especially in the Langmuir model, were too high, invalidating the adjustment in those cases, so the Freundlich’s model shows the best results.

**Figure 2:**
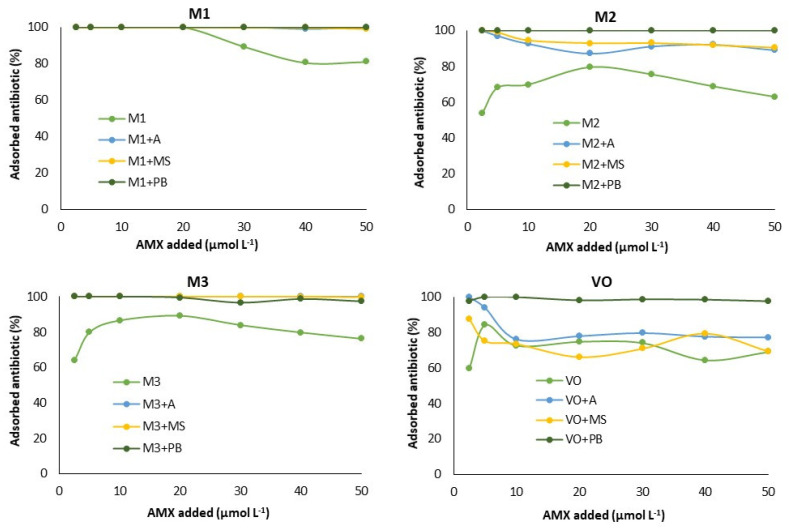
Adsorbed antibiotic (%) for each soil and the mixtures soil + bio-adsorbent in relation to the concentration of AMX added. M: maize soil; VO: vineyard soil; A: wood ash; MS: mussel shell; PB: pine bark; AMX: amoxicillin. Average values (*n* = 3), with coefficients of variation always <5%.

Figure 2 shows the AMX adsorption results (expressed as percentage values) for the different soils with or without bio-adsorbents. It is evident that, in general, adsorption is lower in the vineyard soil, and in the four soils studied, the amount of AMX adsorbed increases when amending with the bio-adsorbents, especially for the three highest concentrations of antibiotic added (30, 40 and 50 µmol L^−1^). In three of the soils (VO, M2 and M3), adsorption increases when amending with the bio-adsorbents, and this takes place for any of the AMX concentrations added; however, in soil M1, this increase occurs just for the three highest concentrations of antibiotic, because for lower concentrations the soil adsorbs 100% of the added antibiotic. These graphs show that the greatest increases in adsorption occur in both VO and M2 soils, especially after the addition of pine bark, while in soils M1 and M3, no differences were found regarding adsorption onto the different bio-adsorbents (Figure 2).

### 3.2. Desorption

Table 3 shows the values of AMX desorption from the different soils depending on the concentration of antibiotic added and the bio-adsorbent used. In general, the higher the concentration of antibiotic added, the greater the desorption from soils, both with and without bio-adsorbent amendments. In some soils, this progressive increase is observed up to 40 µmol L^−1^ of AMX added, with further increase being very scarce or null from this concentration up to 50 µmol L^−1^. In most cases, desorption was lower in soils with one bio-adsorbent than in soils without bio-adsorbents.

## 4. Discussion

### 4.1. Adsorption

In the current study pine bark (with pH 3.99) generally causes an acidification of the amended soil. In this regard, it must be noted that greater acidification is associated with more pronounced AMX adsorption increases, as occurs in soils VO and M2. In previous studies, Githinji et al. [61] found a decrease in AMX adsorption as pH increased from 3.5 to 8.5, whereas other researchers also described a decrease in adsorption for pH values > 5, using pistachio shell [62] or activated carbon [63] as adsorbents.

In the current piece of research, the pH of the soils is above 5, and it was expected that lowering it by incorporating acid adsorbents would facilitate AMX adsorption. In this regard, it is worth noting that, depending on the environmental acid-base conditions, most antibiotics can behave as cations, anions or zwitterions [64], and in the case of AMX the electrical charge of the molecule changes depending on the pH, associated with the charge density present in different functional groups. For AMX, when the pH is lower than its pk_a1_ value (2.98), the amine groups are protonated and the molecule acquires a positive charge; when the pH value is between pk_a1_ (2.98) and pk_a2_ (7.4), the molecule behaves like a zwitterion, with deprotonated carboxyl groups (negative charge density) and protonated amine groups (positive charge density); on the other hand, at pH values between pk_a2_ (7.4) and pk_a3_ (9.6), deprotonated carboxyl and amine groups predominate (with negative charge density); and, finally, at pH > pk_a3_ the phenolic groups are also deprotonated, and the charge is even more negative [65]. When soil pH decreases due to amending with acidic bio-adsorbents (such as pine bark), more positive charges appear on the variable-charge components of those soils, which are summed to those present on the bio-adsorbents, thus being able to bind functional groups of AMX with negatively charged sites, due to electrostatic interactions.

In the current study, the wood ash amendment increased the pH of all soils, while the addition of mussel shell clearly increased the pH of only two of them (M2 and M3) (Table 1). However, an increase in AMX adsorption was also observed with these two amendments (wood ash and mussel shell), similarly to what was achieved when pine bark was added. This is due to the fact that the increase in pH derived from the addition of wood ash and mussel shell causes the appearance of a large number of negative charges in organic matter and in the non-crystalline minerals of the soil, which are very abundant in the soils of this study (Table S1, Supplementary Materials), to be summed to the fact that non-crystalline minerals are also very abundant in wood ash (Table S2, Supplementary Materials). Thus, these negative charges present in the soils and in the bio-adsorbents will facilitate bonds with deprotonated carboxyl groups of the AMX molecule, stablished through cationic bridges (in which Ca probably plays an important role, given its abundance both in wood ash and in mussel shell, Table S2, Supplementary Materials). However, it should be noted that, in the VO soil, despite the fact that wood ash and mussel shell increase the pH, the increase in AMX adsorption is clearly lower than that achieved by amending with pine bark, because organic matter and non-crystalline mineral contents are much lower in this soil than in the other three.

Regarding the fittings to adsorption models, starting with the Linear model the values of the distribution coefficient (*K_d_)* were in the range between 1 and 1525.76 L kg^−1^ in maize soils, and between 5.93 and 112.34 L kg^−1^ in the vineyard soil (Table 2). These values are lower than those reported in previous studies for tetracycline antibiotics [54], but are higher than for sulfonamides [66]. This would indicate that the interactions with soils that give rise to AMX adsorption are weaker than those taking place with tetracycline antibiotics, but stronger than those affecting sulfonamides. Regarding the Freundlich model, the *K_F_* values (affinity coefficient, related to adsorption capacity) vary between 1 and 139.24 L^n^ µmol^1-n^ kg^−1^ in maize soils, and between 9.58 and 109.63 L^n^ µmol^1-n^ kg^−1^ in the vineyard soil. These results are also lower than those obtained by other authors [56] for tetracyclines, but higher than those obtained for sulfonamide [64]. As for the Freundlich’s *n* values, in the case of maize soils they are between 0 and 0.926, while in the vineyard soil they range between 0.298 and 0.892. These *n* values are lower than 1 in all soils, which would indicate that adsorption is not linear, coinciding with that obtained by other authors [61] for other materials. In fact, values of *n* < 1 indicate the presence of heterogeneous adsorption sites and a non-linear and concave curve, which means that the number of available adsorption sites decreases when the concentration of the added contaminant increases, occupying firstly the high energy adsorption sites [67,68]. Regarding the Langmuir model, the *K_L_* values range between 0.05 and 103.81 L µmol^−1^ in maize soils, and between 0.037 and 0.975 L µmol^−1^ in vineyard soils (Table 2).

As shown in Figure 1 and in Table S3 (Supplementary Materials), both soils M1 and M3 have very high AMX adsorption scores (sometimes close to 100%) for most of the antibiotic concentrations added. For these two soils, the incorporation of bio-adsorbents causes modifications in adsorption that are lower than the results reached in the other two soils. In the soils that adsorb less AMX (VO and M2), the mixtures with wood ash, mussel shell or pine bark generally continue to present high R^2^ values (>0.80) for the three models (Table 2), but the high errors associated with the estimation of some parameters invalidate the fittings in several cases (especially in the Langmuir model).

### 4.2. Desorption

Regarding desorption, focusing on the maximum concentration added (50 µmol L^−1^), unamended soils desorb between 6% and 17% of the AMX previously adsorbed, while the release of the antibiotic from the mixtures of soil + bio-adsorbent was always lower than 6%. The greatest decrease in desorption occurred in soil M2 when adding pine bark, going from 17% to 2.5%. Similar results were obtained previously for tetracyclines and sulfonamides [56,66], by researchers who added pine bark to different soils, detecting a decrease in desorption of up to 12% for tetracyclines, and up to 17% for sulfonamides. In this regard, a previous study [69] indicated that the presence of tannins in pine bark favors adsorption (and decreases desorption). It should be noted that AMX desorption has been mainly studied in wastewater, while most of the studies on the adsorption of this antibiotic in soils omit desorption processes. In aqueous matrices, the reported AMX desorption values went from 5% when almond shell ashes were added [70] up to 40% in cases where clay materials were used as adsorbents [71].

## 5. Conclusions

The pH and the abundance of non-crystalline minerals and organic matter are the most determining factors in the adsorption processes of the antibiotic amoxicillin (AMX) in the soils evaluated in this study, both alone and amended with the three tested bio-adsorbents (pine bark, wood ash and mussel shell). It was evidenced that AMX adsorption increased when the crop soils used (devoted to maize and vineyard cultivation) were mixed with the different bio-adsorbents. This increase was higher when pine bark (the bio-adsorbent with the most acidic pH) was added. In addition, AMX desorption decreased when the bio-adsorbent materials were incorporated into the soil, reaching values that did not exceed 6%. The overall results obtained in the current research show that, regarding its applicability, the incorporation of the three bio-adsorbents into agricultural soils contaminated by AMX reduced the risk of transport and passage of the antibiotic to surface and groundwater, and therefore to the food chain, which in fact entails important implications for the environment and public health. In future studies, it would be interesting to evaluate the effect of other bio-adsorbents, as well as soils with different characteristics compared to those used here. Furthermore, possible additional studies could delve into the mechanisms that explain the retention and release processes of AMX when it reaches the environment as a pollutant.

## Figures and Tables

**Table 1:** pH values of the different soils and soil + bio-adsorbent mixtures. VO: vineyard soil; M: maize soils; A: wood ash; MS: mussel shell; PB: pine bark. Average values (*n* = 3), with coefficients of variation always <5%.

Soils and Mixtures	pH	Soils and Mixtures	pH
M1	5.33	M3	5.01
M1 + A	6.93	M3 + A	6.93
M1 + MS	5.29	M3 + MS	5.46
M1 + PB	4.92	M3 + PB	4.79
M2	5.65	VO	6.04
M2 + A	7.04	VO + A	7.81
M2 + MS	5.76	VO + MS	5.92
M2 + PB	5.24	VO + PB	5.35

**Table 2:** Values of the adsorption parameters corresponding to the Freundlich (*K_F_*, expressed in L^n^ µmol^1-n^kg^−1^, and *n*–dimensionless-), Langmuir (*K_L_*, expressed in L µmol^−1^, and *q_m_* -µmol kg^−1^-) and Linear (*K_d_*, expressed in L kg^−1^) models. M: maize soil; VO: vineyard soil; A: wood ash; MS: mussel shell; PB: pine bark; --: fitting not possible.

	Freundlich					Langmuir					Linear		
Sample	*K_F_*	Error	*n*	Error	R^2^	*K_L_*	Error	*q_m_*	Error	R^2^	*K_d_*	Error	R^2^
M1	50.79	34.56	0.274	0.344	0.723	--	--	--	--	--	3.699	0.122	0.983
M1 + A	--	--	--	--	--	0.78	0.209	2066.7	0	0.344	1525.8	358.85	0.344
M1 + MS	139.24	36.56	0.191	0.145	0.745	27.983	29.168	129	30.23	0.746	--	--	--
M1+ PB	--	--	--	--	--	--	--	--	--	--	--	--	--
M2	11.81	4.224	0.676	0.141	0.896	0.074	0.039	140.85	40.68	0.935	5.057	0.568	0.813
M2 + A	31.042	6.881	0.758	0.16	0.932	--	--	--	--	--	22.265	1.671	0.911
M2 + MS	40.022	3.142	0.672	0.062	0.986	0.183	0.064	243.06	53.74	0.984	26.378	1.625	0.939
M2 + PB	91.91	6.984	0.391	0.108	0.923	1.725	0.795	154	27.49	0.934	69.95	11.36	0.633
M3	19.17	4.626	0.67	0.114	0.928	0.124	0.049	161.82	34.48	0.961	3.084	0.113	0.978
M3 + A	--	--	--	--	--	0.05	0	--	--	--	--	--	--
M3 + MS	107.418	7.279	0	0.038	0.978	103.812	287.076	--	--	--	--	--	--
M3 + PB	98.89	11.05	0.282	0.15	0.85	7.342	6.343	112.88	20.25	0.85	94.94	15.65	0.622
VO	9.579	2.155	0.806	0.091	0.974	0.037	0.017	232.67	75.91	0.982	5.934	0.312	0.957
VO + A	10.99	2.246	0.892	0.094	0.982	--	--	--	--	--	8.694	0.313	0.979
VO + MS	10.25	4.764	0.795	0.196	0.893	--	--	--	--	--	6.287	0.571	0.875
VO + PB	109.63	14.06	0.766	0.214	0.815	--	--	--	--	--	112.34	13.83	0.778

**Table 3:** AMX desorption, in µmol kg^−1^ and in percentage between brackets, from the soils studied, with or without bio-adsorbents, as a function of the concentration of antibiotic added (C_0_). M: maize soils; VO: vineyard soil; A: wood ash; MS: mussel shell; PB: pine bark; --: no value. Average values (*n* = 3), with coefficients of variation always <5%.

	C_0_ (µmol L^−1^)						
Sample	2.5	5	10	20	30	40	50
M1	0.349 (10.9)	1.181 (12.5)	2.331 (12.8)	2.819 (11.1)	4.781 (7.2)	4.816 (11.6)	6.21 (16.9)
M1 + A	0 (0)	0 (0)	0 (0)	0.414 (0.79)	1.698 (2.26)	2.482 (2.51)	5.062 (4.10)
M1 + MS	0 (0)	0 (0)	0 (0)	0.233 (0.44)	2.259 (3.05)	3.943 (3.98)	6.105 (4.99)
M1 + PB	0 (0)	0 (0)	0 (0)	0 (0)	0.824 (1.09)	0.834 (0.84)	1.851 (1.50)
M2	0.767 (9.25)	1.339 (12.19)	3.029 (16.12)	5.032 (13.17)	5.211 (6.07)	11.504 (8.07)	18.489 (8.36)
M2 + A	0 (0)	0 (0)	0 (0)	0 (0)	0 (0)	0 (0)	0.935 (0)
M2 + MS	0 (0)	0.047 (0.34)	0.176 (0.77)	--	0.69 (0.99)	1.061 (1.16)	3.488 (3.09)
M2 + PB	--	0.164 (1.13)	0.329 (1.35)	0.713 (1.46)	1.075 (1.46)	1.788 (1.83)	2.597 (2.19)
M3	0.384 (7.45)	0.828 (8.88)	2.6 (10.40)	4.639 (6.11)	--	6.151 (9.33)	6.107 (9.67)
M3 + A	0.283 (3.82)	0.313 (2.45)	--	0.949 (1.80)	1.055 (1.40)	2.319 (2.31)	--
M3 + MS	--	0.258 (2.01)	--	2.488 (4.74)	2.694 (3.58)	--	4.684 (3.79)
M3 + PB	0 (0)	0 (0)	0 (0)	0 (0)	0 (0)	0.14 (0.14)	1.276 (1.05)
VO	0.357 (7.67)	0.735 (13.41)	2.115 (13.85)	2.446 (8.58)	4.741 (8.15)	8.139 (6.26)	8.682 (7.68)
VO + A	0 (0)	0 (0)	0 (0)	0 (0)	0 (0)	0 (0)	0 (0)
VO + MS	0 (0)	0 (0)	0 (0)	0 (0)	0 (0)	0 (0)	0 (0)
VO + PB	0 (0)	0 (0)	0 (0)	0 (0)	0.219 (0.30)	0.712 (0.73)	--

## Data Availability

Not applicable.

## Supplementary Materials

The following supporting information can be downloaded at: https://www.mdpi.com/article/10.3390/ma15093200/s1. Table S1. Values corresponding to the basic parameters determined in the various soils studied. M: maize soils; VO: vineyard soils; OC: organic carbon; OM: organic matter; eCEC: effective cation exchange capacity; Cae, Mge, Nae, Ke, Ale: elements in the exchange complex; o subindex: non-crystalline form; pyr subindex: crystalline form. Average values (*n* = 3), with coefficients of variation always <5%.; Table S2. Characteristics of the bio-adsorbent materials. Cae, Mge, Nae, Ke, Ale: elements in the exchange complex; Sat. Al: Al-saturation in the exchange complex; eCEC: effective cation exchange capacity; XT: total content of the element (X); Alo, Feo: non-crystalline Al and Fe; <LD: below detection level. Average values (*n* = 3), with coefficients of variation always <5%.; Table S3. AMX adsorption, expressed in µmol kg^−1^ (and in percentage between brackets), for the various soils studied, with or without bio-adsorbents, as a function of the concentration of antibiotic added. M: maize (corn) soils; VO: vineyard soils; A: ashes; MS: mussel shell; PB: pine bark. Average values (*n* = 3), with coefficients of variation always <5%.; Figure S1. Example chromatograms corresponding to AMX adsorption onto soils amended with bio-adsorbents
