# Supplementary material for: Amoxicillin Retention/Release in Agricultural Soils Amended with Different Bio-Adsorbent Materials
Source: Materials (Basel). 2022 Apr 28;15(9):3200. doi: 10.3390/ma15093200 (PMC9100866; doi:10.3390/ma15093200)
Supplement: Supplementary file 1 [file materials-15-03200-s001.zip › materials-1673067-supplementary.pdf]

Supplementary material

Amoxicillin retention/release in agricultural soils amended with different bio-adsorbent materials

Raquel Cela-Dablanca[1,*], Ana Barreiro[1], Lucia Rodríguez López[2], Vanesa Santás-Miguel[2], Manuel Arias-Estévez[2], María J. Fernández-Sanjurjo[1], Esperanza Álvarez-Rodríguez[1], Avelino Núñez-Delgado[1]

[1] Department of Soil Science and Agricultural Chemistry, Engineering Polytechnic School, Univ. Santiago de Compostela, 27002 Lugo, Spain
[2] Soil Science and Agricultural Chemistry, Fac. Sciences, Univ. Vigo, 32004 Ourense, Spain

[*] Corresponding author

Characterization of soils and sorbent materials

The values corresponding to pH in water and pH in KCl (solid:liquid ratio 1:2.5) were measured with a CRISON-2001 pHmeter (Crison, Barcelona, Spain). Total C and N were determined by means of elemental analysis, using a LECO CHN-100 equipment (Leco Corporation, St. Joseph, MI, USA). The exchangeable cations (Ca_e, Mg_e, Na_e, K_e and Al_e) were displaced using a 1 M NH_4Cl solution in a 1:10 soil/bio-adsorbent: solution ratio and were then quantified by means of atomic absorption spectrophotometry, using a Perkin Elemer Analyst 200 apparatus (Perkin Elmer, Inc, USA). The effective cation exchange capacity (eCEC) was determined as the sum of these five exchangeable cations. Available phosphorus was extracted using 0.5 M $NaHCO_3$ and quantified using a UV-visible spectrophotometer (UV-1201, Shimadzu, Kyoto, Japan). To determine total non-crystalline Fe and Al, a specific extraction was performed using ammonium oxalate buffered at pH 3, stirred for 4 hours in the dark, then adding 5 drops of 0.25% superfloc to the resulting extract, then centrifuging at 2000 rpm for 10 minutes, filtering and diluting the supernatant to 1:5. Finally, Fe and Al in non-crystalline form (Fe_o and Al_o) were quantified by atomic absorption spectrophotometry. In addition, Fe and Al were determined after extraction with sodium pyrophosphate at pH 10. The extraction was performed shaking 1 g of sample with 100 ml of sodium pyrophosphate during 16 h. Finally, Fe and Al were quantified by atomic absorption spectrophotometry. Total contents of different elements (Na_T, Mg_T, Al_T, K_T, Ca_T, Cr_T, Mn_T, Fe_T, Co_T, Ni_T, Cu_T, Zn_T, As_T and Cd_T) were determined following the EPA 3051 method, performing an acid digestion in a microwave with a 65% HNO_3 solution, and subsequent determination with ICP-MS.

Table S1. Values corresponding to the basic parameters determined in the various soils studied. M: maize soils; VO: vineyard soils; OC: organic carbon; OM: organic matter; eCEC: effective cation exchange capacity; Ca_e, Mg_e, Na_e, K_e, Al_e: elements in the exchange complex; o subindex: non-crystalline form; pyr subindex: crystalline form. Average values (n=3), with coefficients of variation always < 5%

Soil	pH_{water}	OC	OM	N	Sand	Clay	eCEC	Ca_e	Mg_e	Na_e	K_e	Al_e	Fe_o	Fe_{pyr}	Al_o	Al_{pyr}
		%					cmol$_c$ kg^{-1}						mg kg^{-1}			
M1	5.33	2.66	4.59	0.27	43.42	24.65	6.88	5.35	0.67	0.02	0.44	0.40	5745	3159	3401	2871
M2	5.65	2.58	4.44	0.25	43.57	22.43	7.48	6.18	0.77	0.00	0.28	0.24	5780	3315	3881	2717
M3	5.01	2.04	3.52	0.21	45.64	22.43	5.94	4.58	0.67	0.10	0.17	0.43	4545	2923	2896	1945
VO	6.04	1.77	3.06	0.10	49.57	24.14	7.42	4.94	0.86	0.16	0.69	0.77	1790	1171	1556	1141

Table S2. Characteristics of the bio-adsorbent materials. Ca_e, Mg_e, Na_e, K_e, Al_e: elements in the exchange complex; Sat. Al: Al-saturation in the exchange complex; eCEC: effective cation exchange capacity; X_T: total content of the element (X); Al_o, Fe_o: non-crystalline Al and Fe; <LD: below detection level. Average values (n=3), with coefficients of variation always <5%.

Parameter	Unit	Pine bark	Wood ash	Mussel shell
C	%	48.70	13.23	11.43
N	%	0.08	0.22	0.21
C/N		608.75	60.14	55.65
pH_{water}		3.99	11.31	9.39
pH_{KCl}		3.42	13.48	9.04
Ca_e	$cmol_c\ kg^{-1}$	5.38	95.0	24.75
Mg_e	$cmol_c\ kg^{-1}$	2.70	3.26	0.72
Na_e	$cmol_c\ kg^{-1}$	0.46	12.17	4.37
K_e	$cmol_c\ kg^{-1}$	4.60	250.65	0.38
Al_e	$cmol_c\ kg^{-1}$	1.78	0.07	0.03
eCEC	$cmol_c\ kg^{-1}$	14.92	361.15	30.25
Sat Al	%	11.93	0.02	0.10
Available-P	$mg\ kg^{-1}$	70.45	462.83	54.17
Na_T	$mg\ kg^{-1}$	68.92	2950	5174.00
MgT	mg/kg	473.55	26171	980.66
Al_T	$mg\ kg^{-1}$	561.50	14966	433.24
K_T	$mg\ kg^{-1}$	737.84	99515	202.07
Ca_T	$mg\ kg^{-1}$	2318.81	136044	280168
Cr_T	$mg\ kg^{-1}$	1.88	36.28	4.51
Mn_T	$mg\ kg^{-1}$	30.19	10554	33.75
Fe_T	$mg\ kg^{-1}$	169.78	12081	3535
Co_T	$mg\ kg^{-1}$	0.20	17.25	1.02
Ni_T	$mg\ kg^{-1}$	1.86	69.25	8.16
Cu_T	$mg\ kg^{-1}$	<LD	146.33	6.72
Zn_T	$mg\ kg^{-1}$	6.98	853.00	7.66
As_T	$mg\ kg^{-1}$	<LD	8.36	1.12
Cd_T	$mg\ kg^{-1}$	0.13	19.93	0.07
Al_o	$mg\ kg^{-1}$	315.0	8323	178.33
Fe_o	$mg\ kg^{-1}$	74.0	4233	171.0

Table S3. AMX adsorption, expressed in µmol kg^{-1} (and in percentage between brackets), for the various soils studied, with or without bio-adsorbents, as a function of the concentration of antibiotic added. M: maize (corn) soils; VO: vineyard soils; A: ashes; MS: mussel shell; PB: pine bark. Average values (n=3), with coefficients of variation always < 5%

Soil	AMX added (µmol/L)	AMX adsorbed, concentration and %	Soil	AMX added (µmol/L)	AMX adsorbed, concentration and %
M1	0	0 (0)	M2	0	0 (0)
	2.5	7.02 (100)		2.5	4.15 (53.60)
	5	10.73 (100)		5	6.79 (68.07)
	10	23.67 (100)		10	16.12 (69.80)
	20	45.20 (100)		20	35.22 (79.57)
	30	70.05 (89.37)		30	59.63 (75.47)
	40	88.02 (80.57)		40	76.21 (68.76)
	50	94.87 (81.07)		50	73.08 (62.88)
M1+A	0	0 (0)	M2+A	0	0 (0)
	2.5	7.42 (100)		2.5	6.76 (100)
	5	12.71 (100)		5	12.91 (97.03)
	10	25.99 (100)		10	24.03 (92.60)
	20	52.30 (100)		20	43.36 (87.15)
	30	75.01 (100)		30	67.99 (91.11)
	40	98.83 (98.82)		40	91.69 (92.14)
	50	123.33 (99.85)		50	110.99 (89.01)
M1+MS	0	0 (0)	M2+MS	0	0 (0)
	2.5	7.33 (100)		2.5	6.32 (100)
	5	12.71 (100)		5	13.94 (98.98)
	10	26.01 (100)		10	22.99 (94.51)
	20	52.44 (100)		20	46.24 (92.94)
	30	74.14 (99.85)		30	69.37 (92.96)
	40	99.17 (99.67)		40	91.38 (91.84)
	50	122.17 (98.92)		50	113.03 (90.42)
M1+PB	0	0 (0)	M2+PB	0	0 (0)
	2.5	7.34 (100)		2.5	7.34 (100)
	5	12.88 (100)		5	12.88 (100)
	10	26.07 (100)		10	26.07 (100)
	20	52.76 (100)		20	52.76 (100)
	30	75.74 (100)		30	75.74 (100)
	40	99.62 (100)		40	99.62 (100)
	50	123.37 (100)		50	123.37 (100)

Soil	AMX added (µmol/L)	AMX adsorbed, concentration and %	Soil	AMX added (µmol/L)	AMX adsorbed, concentration and %
	0	0 (0)		0	0 (0)
	2.5	4.80 (63.96)		2.5	4.54 (59.82)
	5	8.28 (80)		5	8.81 (84.22)
M3	10	20.33 (86.63)	VO	10	16.84 (72.64)
	20	40.04 (89.23)		20	32.87 (74.87)
	30	65.98 (83.93)		30	58.64 (74.16)
	40	87.21 (79.77)		40	76.89 (64.45)
	50	89.74 (76.21)		50	80.81 (69.06)
	0	0 (0)		0	0 (0)
	2.5	7.41 (100)		2.5	6.72 (100)
	5	12.78 (100)		5	12.30 (93.94)
M3+A	10	26.21 (100)	VO+A	10	19.94 (76.04)
	20	52.66 (100)		20	38.77 (77.93)
	30	75.50 (100)		30	59.17 (79.68)
	40	100.40 (100)		40	77.24 (77.63)
	50	123.74 (100)		50	96.23 (77.16)
	0	0 (0)		0	0 (0)
	2.5	7.40 (100)		2.5	5.30 (87.65)
	5	12.84 (100)		5	9.96 (75.26)
M3+MS	10	25.96 (100)		10	19.33 (73.60)
	20	52.52 (100)		20	32.95 (66.22)
	30	75.20 (100)	VO+MS	30	52.96 (70.96)
	40	99.92 (99.92)		40	79.10 (79.50)
	50	123.59 (99.57)		50	85.75 (69.46)
	0	0 (0)		0	0 (0)
	2.5	7.04 (100)		2.5	5.93 (97.75)
	5	14.16 (100)		5	13.15 (100)
M3+PB	10	26.26 (100)		10	25.92 (99.97)
	20	49.73 (99.45)	VO+PB	20	49.03 (98.05)
	30	72.48 (96.64)		30	73.92 (98.57)
	40	98.65 (98.65)		40	97.94 (98.43)
	50	121.41 (97.34)		50	120.44 (97.49)

Quantification of AMX

The HPLC determinations were carried out with a Model LPG 3400 SD equipment (Thermo-Fisher, USA). Some example chromatograms are included in Figure S1.

For HPLC quantifications, a Luna C18 column (150 mm long, 4.6 mm internal diameter, 5 μm particle size) was used, being provided by Phenomenex (Madrid, Spain), as well as a pre-column (4 mm long, 2 mm in diameter, 5 μm particle size) packed with the same material as the column. The injection volume was 50 μL and the flow rate was 1.5 ml min^{-1}. The mobile phase consisted of acetonitrile (phase A) and 0.01 M phosphoric acid (phase B). A linear gradient was used varying from 5% to 15% of phase A and from 95% to 85% of phase B. The initial conditions were re-established in 2 min and maintained for 2 min. The total analysis time was 10 min, with a retention time of 3.3 min, and the wavelength used for detection was 230 nm. The amounts of antibiotic adsorbed were calculated by the difference between the concentrations initially present in the added solutions and those remaining in the solutions at equilibrium. All determinations were made in triplicate.

M3 soil with pine bark amendment; AMX concentration added 50 μmol $L^{-1.}$

M2 soil with mussel shell amendment, AMX concentration added 20 μmol L^{-1}

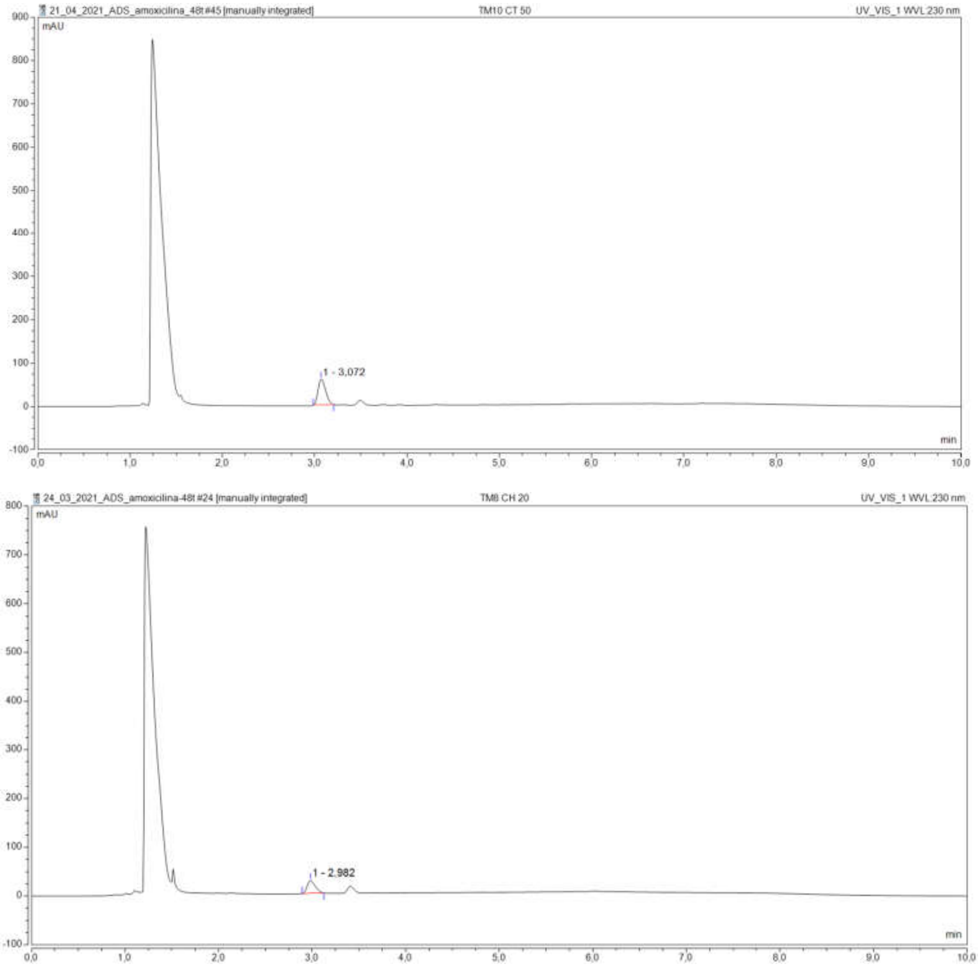

Figure S1. Example chromatograms corresponding to AMX adsorption onto soils amended with bio-adsorbents.
